# Routine preoperative mechanical bowel preparation with additive oral antibiotics is associated with a reduced risk of anastomotic leakage in patients undergoing elective oncologic resection for colorectal cancer

**DOI:** 10.1186/s12957-019-1563-2

**Published:** 2019-01-16

**Authors:** Peter C. Ambe, Konstantinos Zarras, Maciej Stodolski, Ingfu Wirjawan, Hubert Zirngibl

**Affiliations:** 1 Helios University Hospital Wuppertal, Witten/Herdecke University, Heusnerstr. 40, 42283 Wuppertal, Germany; 2 Department of Visceral, Minimally Invasive and Oncologic Surgery, Marien Hospital Düsseldorf, Rochusstr 2, 40479 Düsseldorf, Germany

## Abstract

**Background:**

Anastomotic leakage (AL) following colorectal resection is a serious issue. AL in oncologic patients might negatively affect the overall survival. Recently, mechanical bowel preparation with additive oral antibiotics (MBP + AB) prior to surgery has been suggested as a means of reducing AL. However, it is unclear whether this positive effect is secondary to MBP alone or secondary to the additive oral antibiotic (MBP + AB). The aim of this study was to investigate the effect of mechanical bowel preparation with additive oral antibiotics (MBP + AB) and without additive oral antibiotics (MBP − AB) on the rate of AL following colorectal resection for cancer.

**Materials and methods:**

Patients undergoing surgical management for colorectal cancer with anastomosis from January 2014 till September 2017 were included for analysis. Cases undergoing MBP + AB were included in the study group. Patients undergoing MBP − AB were included in the control group. Both groups were compared with regard to the rate of AL.

**Results:**

Four hundred and ninety-six patients: 125 undergoing MBP + AB and 371 undergoing MBP – AB were included for analysis. Significantly, more male patients were included in the MBP – AB group compared to the MBP + AB group: 60.1% vs. 45.6% respectively (*p* = 0.03). Both groups were similar with regard to age distribution and clinicopathological findings (*p* > 0.05). The rate of AL was significantly higher in the control group (MBP − AB) compared to study group (MBP + AB) (9.1% vs. 4.0%, *p* = 0.03).

**Conclusion:**

Mechanical bowel preparation with additive oral antibiotics prior to elective colorectal resection with anastomosis significantly reduces the risk of AL. Therefore, mechanical bowel preparation with additive non-absorbable oral antibiotics should be recommended in all cases prior to elective colorectal surgery.

## Background

Colorectal cancer (CRC) is a common problem, and oncologic resection remains the only means of cure. Anastomotic leakage (AL) following resection of colon and rectal cancer represents the most feared complication [1, 2]. This single complication is a leading cause of significant morbidity and mortality following radical surgery for CRC. Anastomotic leakage might be associated with dalliance or omission of chemotherapy [3]. More so, AL has been shown to be associated with an increased risk of local cancer recurrence [4]. These factors have been shown to be associated with reduced overall survival [5, 6]. The effect of mechanical bowel preparation (MBP) prior to colorectal resection on the risk of AL has been controversial in the past with constantly changing recommendations [7, 8]. Recently, many studies have identified a reduced risk of AL following preoperative MBP with additive oral antibiotics [9]. It is unclear whether the reduced risk of AL is primarily due to MBP or is secondary to oral antibiotics. This study was designed to study the effect of preoperative MBP with and without oral antibiotics on the rate of AL in patients undergoing oncologic colorectal resection with anastomosis for CRC.

## Materials and methods

This study was approved by the Institutional Review Board at the Witten/Herdecke University. This is a retrospective analysis of prospectively collected data of patients undergoing oncologic resection of colorectal cancer. The data of all consecutive patients diagnosed with CRC is prospectively put into an institutional database by trained study nurses and research fellows as reported elsewhere [10, 11]. The database is continuously updated with data on the present status of all registered patients. All patients diagnosed with CRC are discussed in an interdisciplinary oncologic board prior to surgery. Oncologic colorectal surgery included central dissection of the mesenteric lymphovascular pathways in accordance with complete mesocolic excision (CME) in cases with colon cancer as described by Hohenberger et al. [12], partial mesorectal excision (PME) for proximal rectal cancer and cancer of the rectosigmoidal junction, and total mesorectal excision (TME) in cases with mid and low rectal cancer as described by Heald et al. [13]. Data of patients undergoing oncologic colorectal resection between January 2014 and September 2017 were included for analysis.

MBP was performed in all cases prior to surgery. In the period prior to May 2016, preoperative MBP was performed using either polyethylenglykol or sodium–magnesium sulfate 24 h prior to surgery. Beginning June 2016, oral antibiotics (1 g vancomycin and 400 mg metronidazole) per liter PEG were added to the above preparation. Our study group included all cases managed after May 2016, i.e., patients who underwent MBP with additive oral antibiotics while all cases undergoing MBP without oral antibiotics were included in the control group. Perioperative single shot antibiotics was given to all patients.

The extent of CRC was classified using both the American Joint Committee on Cancer (AJCC) TNM and Union for International Cancer Control (UICC) staging systems following histopathology in all cases [14]. Patient’s demographic information including sex and age at the time of diagnosis were recorded. Cancer specific parameters including the tumor stage (pT), nodal involvement (pN), the presence or absence of distance metastases (pM), and tumor location were noted. All patients undergoing oncologic resection were included for analysis. Exclusion criteria included: palliation procedures, patients with multiple cancers, patients undergoing emergency surgery, and cases without anastomosis.

The main endpoint was the rate of AL. AL was suspected following clinical presentation and findings from physical examination. The diagnosis was confirmed using contrast-enhanced computed tomography, endoscopic examination, or during surgery. The extent of AL was characterized using the grading system proposed by Rahbari et al. [15]. According to this grading system, AL requiring no therapeutic intervention is classified as grade A and grade B leakages are managed via endoscopic, sonographic, or radiologic intervention, while grade C leakages warrant surgical revision.

## Statistics

The Statistical Package for Social Science (SPSS) version 24 (IBM Corp, Armonk, NY, USA) was used for data analysis. The study population was described using median and interquartile ranges where necessary. The chi square test and Mann-Whitney *U* test were employed for analytic statistics and odds ratio calculation. The two-sided *p* values were reported where necessary with the level of significance set as *p* < 0.05. A 95% confidence interval was used in all statistical analyses.

## Results

**Fig. 1:**
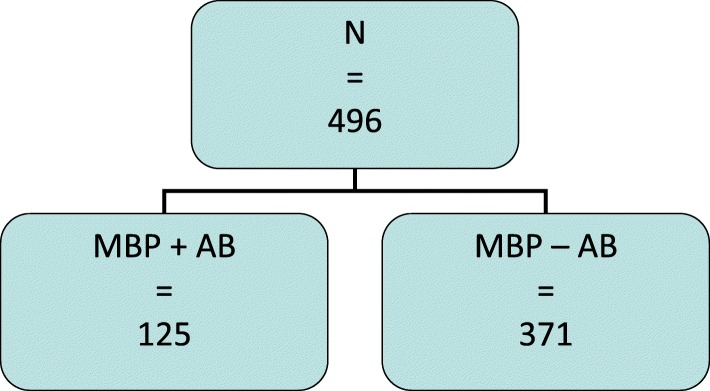
Distribution of the study population. Patients undergoing MBP with oral antibiotics were included in the study group, while the control group included patients undergoing MBP without oral antibiotics

Four hundred and ninety-six patients underwent oncologic colorectal resection with anastomosis for CRC within the period of investigation. Colon cancer was managed in 370 cases while rectal cancer was managed in 126 cases. MBP with additive oral antibiotics (study group) was performed in 125 cases including 68 (54.4%) females and 57 (45.6%) males. MBP without oral antibiotics (control group) was done in 371 cases including 148 (39.9%) females and 223 (60.1%) males (Fig. 1). Significantly, more male patients were included in the group without oral antibiotics compared to the group with oral antibiotics: 60.1% vs. 45.6% respectively, *p* = 0.03. However, sex could not be identified as a risk factor for AL on multivariate analysis.

The median age of the study group was 73.0 years (range 21–91 years) while the median age of the control group was 70.0 years (range 31–92 years). There was no statistically significant difference amongst both groups with regard to age (*p* = 0.413). The clinicopathologic findings in this study are presented in Table 1.

**Table 1:** Baseline and clinical characteristics of the study population, AJCC

Characteristics	MBP + AB	MBP − AB	*p* value
N	125	371	
Sex			
Female	69 (55.2%)	148 (39.9%)	0.03
Male	56 (44.8%)	223 (60.1%)	
Median age	73.0 years	70.0 years	0.10
Range	21–91 years	31–92 years	
Location			
Right colon	62 (49.6%)	138 (37.2%)	0.06
Left colon	34 (27.2%)	94 (25.3%)	
Rectum	29 (22.2%)	139 (37.5%)	
AJCC tumor stage (pT)			
1	11 (8.80%)	42 (11.3%)	*p* > 0.05
2	24 (19.2%)	81 (21.8%)	
3	70 (56.0%)	191 (51.5%)	
4	20 (16.0%)	57 (15.4%)	
AJCC nodal stage (pN)			
0	79 (63.2%)	211 (56.9%)	*p* > 0.05
1	29 (23.2%)	105 (28.3%)	
2	17 (13.6%)	55 (14.8%)	
UICC			
I	28 (22.4%)	96 (25.8%)	*p* > 0.05
II	47 (37.6%)	106 (28.6%)	
III	33 (26.4%)	103 (27.8%)	
IV	17 (13.6%)	66 (17.8%)	

There was no statistically significant difference amongst both groups with regard to the AJCC and UICC cancer stages. Equally, there was no statistically significant difference amongst both groups with regard to cancer location (Table 1).

**Fig. 2:**
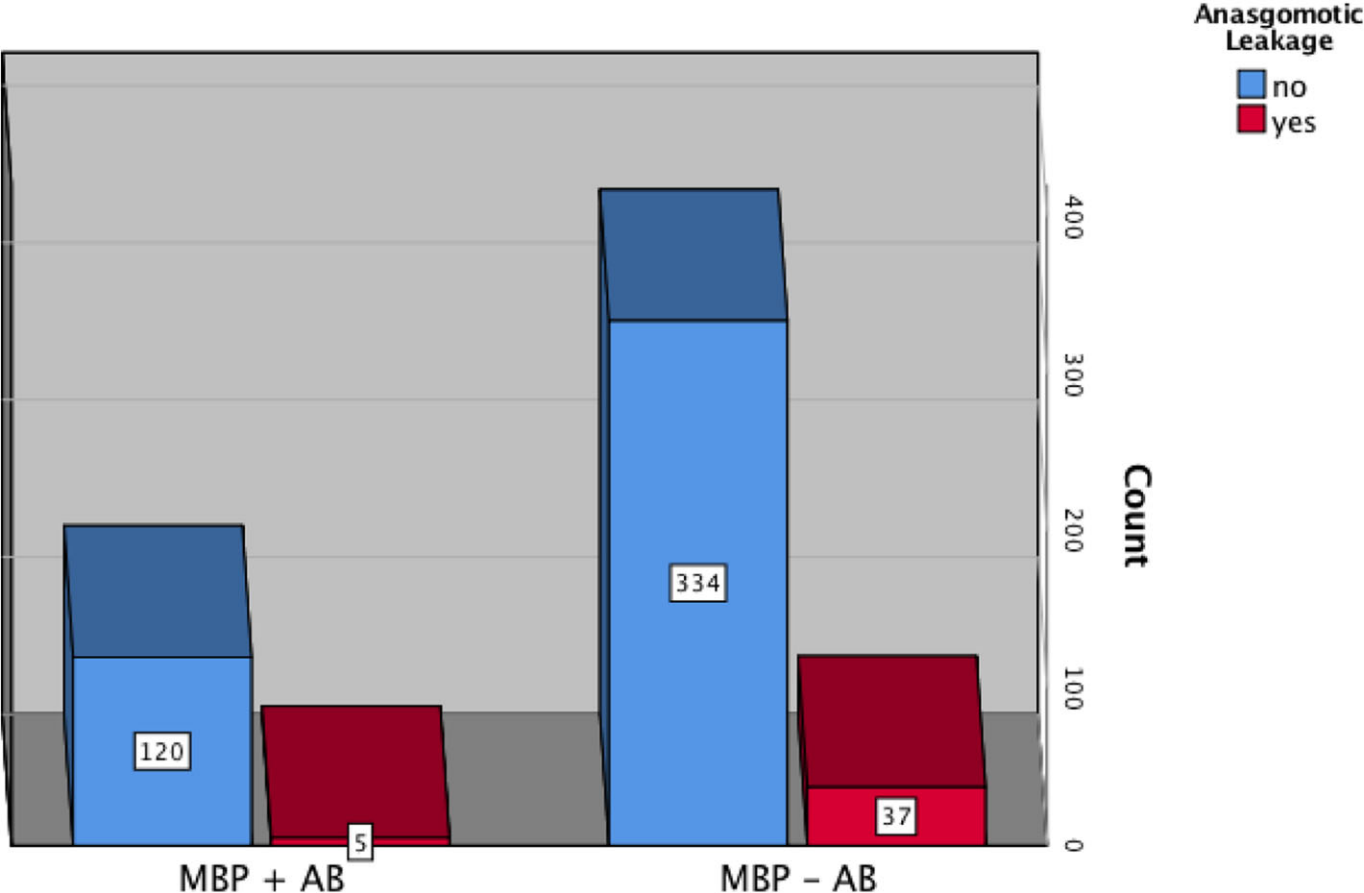
Rates of AL in both groups. A significantly higher rate of AL was recorded following MBP without oral antibiotics compared to the group with additive oral antibiotics

Forty-two cases of AL (8.5%), including three grade A, six grade B, and 33 grade C, were recorded in this series (Table 2). In 25 cases, AL occurred after colon resection (25/370 = 6.7%) and in 17 cases following rectal resection (17/126 = 13.5%). Five cases (5/125 = 4.0%) of AL were recorded in the study group, four cases (4/95 = 4.2%) after colon resection, and one case (1/30 = 3.3%) after rectal resection. On the other hand, the rate of AL was 10% (37/371) in the control group. This corresponded to 21 cases (21/232 = 9.1%) following colon resection and 16 cases (16/139 = 11.5%) following rectal resection. The rate of AL was significantly higher in the group without additive oral antibiotics (*p* = 0.038, Fig. 2). The risk of AL was more than twice higher in patients undergoing MBP without oral antibiotics was (OR 2.22, 0.96–5.12, p = 0.038).

**Table 2:** Summary of AL in both groups

Characteristics	MBP + AB	MBP − AB	*p* value
Anastomotic leakage	5	37	0.03
Grade A	0	3	
Grade B	0	6	
Grade C	5	28	
AJCC nodal stage (pT)			
1	0	3	
2	0	9	
3	4	18	
4	1	7	
AJCC nodal stage (pN)			
0	5	23	
1	0	9	
2	0	5	
AJCC nodal stage (pM)			
0	5	29	
1	0	8	
UICC			
1	0	10	
2	5	13	
3	0	10	
4	0	4

Amongst all patients undergoing rectal resection, preoperative radiation and chemotherapy were performed in 81 cases. In this group, MBP without oral antibiotics was performed in 71 cases while MBP with oral antibiotics was done in 10 cases. While no case of AL was recorded in patients undergoing MBP with oral antibiotics, six cases of AL were recorded in patients following MBP without oral antibiotics.

## Discussion

The effect of preoperative MBP with and without oral antibiotics on the rates of AL was investigated in this study. A single-center retrospective analysis of prospectively collected data of patients undergoing oncologic colorectal resection for CRC was performed. Preoperative bowel prepping with oral antibiotic adjunct led to a significantly lower rate of AL in comparison to cases following MBP without oral antibiotics.

Clinical practice with regard to preoperative bowel preparation prior to elective colorectal resection has been conflicted. Nichols and Condon reported in 1971 that MBP was associated with decreased morbidity and mortality following colorectal surgery [16]. In contrast, the dogmatic practice of MBP in colorectal surgery was questioned by Irving and Scrimgeour in later years [8]. This trend was supported by results of two meta-analyses by Slim et al. in 2004 and 2009 which failed to show a reduction in the risk of AL following MBP [17, 18]. Thus, the tenor was to abandon MBP prior to colorectal surgery. However, combining oral antibiotics to MBP has been shown to be associated with a reduced rate of AL in recent years [19, 20].

The rate of AL in our study was significantly lower in the group with MBP with oral antibiotics compared to the group without oral antibiotics. As expected, the rate of AL was higher in patients undergoing rectal resection compared to those undergoing segmental colectomy. The risk of AL was 2.2 times higher in patients undergoing MBP without oral antibiotics compared to the group with additive oral antibiotics. This finding suggests that MBP alone might not have any positive influence on the risk of AL. This trend is in accordance with results from a recently published meta-analysis by Rollins et al. [21]. The reduced rate of AL recorded in the group with additive oral antibiotic to MBP in this series is in accordance with current literature [22, 23].

Postoperative morbidity and mortality, especially in the oncologic setting, represent primary outcome measures in patients undergoing colorectal surgery. Anastomotic leakage constitutes a relevant surgical complication, which might negatively influence the overall survival. Therefore, preventing such devastating complications besides adequate oncologic resection with clear margins must be seen as a major postoperative endpoint. Preoperative MBP prior to colorectal resection, especially in the era of laparoscopic surgery, enables a better bowel handling during the dissection and creation of the anastomosis. Besides, the risk of anastomotic disruption via hard stool and subsequently AL is reduced following bowel cleansing. Adding oral non-absorbable antibiotics reduces bacterial bowel colonization, which might influence the rate of infectious complications like AL.

The effects seen in this study cannot be attributed to patient and disease factors since both groups were grossly compatible. Therefore, the reason for this observation must be secondary to reduced bacterial load following the use of oral antibiotics during preoperative MBP.

This study is limited by the retrospective study design and the relatively small size of the study population. This is especially true with regard to the number of patients undergoing rectal resection with or without neoadjuvant chemoradiation. Despite these limitations, the results of this study support the routine use of mechanical bowel prepping with oral antibiotic adjunct prior to elective colorectal surgery.

## Conclusion

Mechanical bowel preparation with additive oral antibiotics prior to elective colorectal resection with anastomosis significantly reduces the risk of AL. Therefore, mechanical bowel preparation with additive non-absorbable oral antibiotics should be recommended in all cases prior to elective colorectal surgery.

## Abbreviations

AL: Anastomotic leakage
CME: Complete mesocolic excision
CRC: Colorectal cancer
MBP: Mechanical bowel preparation
PEG: Polyethylene glycol
PME: Partial mesorectal excision
TME: Total mesorectal excision
